# Complete Genome Sequence of Phage-Like Plasmid pECOH89, Encoding CTX-M-15

**DOI:** 10.1128/genomeA.00356-14

**Published:** 2014-04-24

**Authors:** Linda Falgenhauer, Yancheng Yao, Moritz Fritzenwanker, Judith Schmiedel, Can Imirzalioglu, Trinad Chakraborty

**Affiliations:** a Institute of Medical Microbiology, Justus-Liebig-University Giessen, Giessen, Germany; b German Center for Infection Research (DZIF), Partner Site Giessen-Marburg-Langen, Campus Giessen, Giessen, Germany

## Abstract

A nonconjugative and nontypable plasmid of a clinical *Escherichia coli* isolate expressing resistance to extended-spectrum cephalosporins (ESCs) was isolated and sequenced. The plasmid pECOH89 contains a CTX-M-15 resistance cassette and comprises 111,741 bp, with strong homology to bacteriophage-like plasmids and to the *Salmonella*-specific bacteriophage SSU5.

## GENOME ANNOUNCEMENT

Resistance to extended-spectrum cephalosporins (ESCs) is among the most important resistance determinants spreading worldwide in *Enterobacteriaceae* (1, 2). Plasmids containing resistance genes against ESCs from *Enterobacteriaceae* comprise many different Inc types and those not typeable with Inc schemes (1). The involvement of bacteriophages in the spread of ESC resistance genes has been shown (3).

From a wound swab sample from an inpatient, we isolated the multiresistant *Escherichia coli* strain H89, which harbored *bla*_CTX-M-15_ on a plasmid that was untypeable using current typing schemes (4) and nonconjugative (5). We performed whole-plasmid sequencing to determine the nature of the plasmid type involved.

Plasmid DNA was isolated using Qiagen Maxiprep (Qiagen, Germany) according to the manufacturer’s instructions. Pyrosequencing of a 3-kb mate-paired sequence library was performed on a 454 GS-FLX platform with Titanium chemistry (Vertis, Germany). Reads were assembled *de novo* with Newbler (version 2.5.3, Roche, Germany) to one contig (average coverage of 17-fold). Gap closure was performed using PCR followed by sequencing with ABI BigDye 3.0 technology (Life Technologies, USA). First-pass annotation was performed using RAST (6), and the annotation was subsequently manually curated using ARTEMIS (7). Comparative analysis of data was done using GECO (8). PHAST and tRNAscan were used to identify bacteriophage genes and tRNA genes, respectively (9, 10).

The complete sequence of pECOH89 is 111,741 bp, with a G+C composition of 46.1%. The plasmid was predicted to harbor 128 protein coding sequences (CDS) and two tRNAs for threonine and asparagine. Genomic analysis revealed that pECOH89 has strong homology to the recently sequenced plasmid p09EL50 from an *E. coli* O104:H4 strain isolated in 2009 (GenBank accession no. CP003298) and the *E. coli* strain LF82 plasmid (CU638872), sharing identity of almost 100% for 71 CDS, including *dnaE*, *dnaG*, *parA*, *parB*, *rnhA*, *dam, dfrA*, and *nrdAB*, and of >80% for a further 42 CDS. The replication protein RepA is identical to that found in p09EL50 and similar to that of pLF82 (100% and 48%, respectively). Strong homology was also detected to the cryptic plasmid pHCM2 from *Salmonella enterica* subsp. *enterica* serovar Typhi strain CT18 (accession no. AL513384), as well as to the *Salmonella*-specific bacteriophage SSU5 (accession no. JQ965645). In total, 109 of the 128 CDS were found to be homologous, with >65% identity to bacteriophage genes such as CDS for phage tail fiber, shaft, coat, and terminase, while 19 encoded nonphage proteins. The CTX-M-15 resistance cassette, which is unique to pECOH89 and is absent in the other homologous plasmids, comprised four CDS and included an IS*Ecp1* transposase gene, a gene encoding a hypothetical protein, *bla*_CTX-M-15_, and the putative *orf477*Δ. No other resistance genes were detected. The resistance cassette was identical to that of a clinical *E. coli* O25b-B2-ST131 isolate from Japan (accession no. AB701567.1). Of the remaining 15 nonphage proteins (all annotated as hypothetical), 13 were found as protein sequences or as DNA sequences (not annotated as proteins) in at least one of the plasmid reference genomes (pHCM2, p09EL50, or LF82).

In conclusion, we present data for a novel location of the CTX-M-15 resistance cassette on an atypical extrachromosomal genetic element. The plasmid pECOH89 is a new member of an emerging family of phage-like plasmids.

### Nucleotide sequence accession number.

The whole-genome sequence of pECOH89 has been deposited in ENA under the accession number HG530657.
